# Drug-free remission of immune thrombocytopenic purpura following resection of perihilar cholangiocarcinoma: a case report

**DOI:** 10.1093/jscr/rjae607

**Published:** 2024-09-26

**Authors:** Masayoshi Sakuma, Daigoro Takahashi, Keitaro Kamei, Yuichi Takayama, Takamasa Takahashi, Hiroki Aoyama, Takahiro Hosoi, Atsuyuki Maeda

**Affiliations:** Department of Surgery, Ogaki Municipal Hospital, 4-86, Minaminokawa‐cho, Ogaki city, Gifu, Japan; Department of Surgery, Ogaki Municipal Hospital, 4-86, Minaminokawa‐cho, Ogaki city, Gifu, Japan; Department of Surgery, Ogaki Municipal Hospital, 4-86, Minaminokawa‐cho, Ogaki city, Gifu, Japan; Department of Surgery, Ogaki Municipal Hospital, 4-86, Minaminokawa‐cho, Ogaki city, Gifu, Japan; Department of Surgery, Ogaki Municipal Hospital, 4-86, Minaminokawa‐cho, Ogaki city, Gifu, Japan; Department of Surgery, Ogaki Municipal Hospital, 4-86, Minaminokawa‐cho, Ogaki city, Gifu, Japan; Department of Surgery, Ogaki Municipal Hospital, 4-86, Minaminokawa‐cho, Ogaki city, Gifu, Japan; Department of Surgery, Ogaki Municipal Hospital, 4-86, Minaminokawa‐cho, Ogaki city, Gifu, Japan

**Keywords:** immune thrombocytopenic purpura, perihilar cholangiocarcinoma, hepatopancreatoduodenectomy

## Abstract

Immune thrombocytopenic purpura (ITP) is a rare autoimmune disorder. Although secondary ITP, caused by various underlying diseases, including some malignant tumors, has been reported, instances of ITP resolving after successful treatment of the underlying cause are uncommon and noteworthy. Herein, we present a case of a patient with perihilar cholangiocarcinoma (pCCA) and ITP who achieved drug-free remission of ITP following tumor resection. A 76-year-old man presented with pCCA complicated by ITP. Prednisolone treatment successfully managed his thrombocytopenia, allowing for a left hepatopancreatoduodenectomy. ITP relapse did not occur after discontinuation of prednisolone postoperatively. This case suggests that surgical resection of the underlying malignancy may induce remission of secondary ITP associated with pCCA.

## Introduction

Immune thrombocytopenic purpura (ITP), formerly known as idiopathic thrombocytopenic purpura [1], is a rare autoimmune disorder characterized by the destruction of peripheral platelets and inappropriate production of bone marrow [2]. ITP can further be classified into two main categories: primary ITP, occurring without an underlying disease and secondary ITP caused by various underlying diseases [3]. In ~80% of adult patients with ITP, first-line corticosteroids are ineffective or lead to long-term dependence, necessitating alternative therapies [4]. However, some reports have demonstrated that secondary ITP due to malignant tumors may be resolved with successful treatment of the underlying cause [5–7].

Herein, we report a case of a patient with perihilar cholangiocarcinoma (pCCA) and ITP, who achieved drug-free remission of ITP following tumor resection.

## Case report

**Figure 1:**
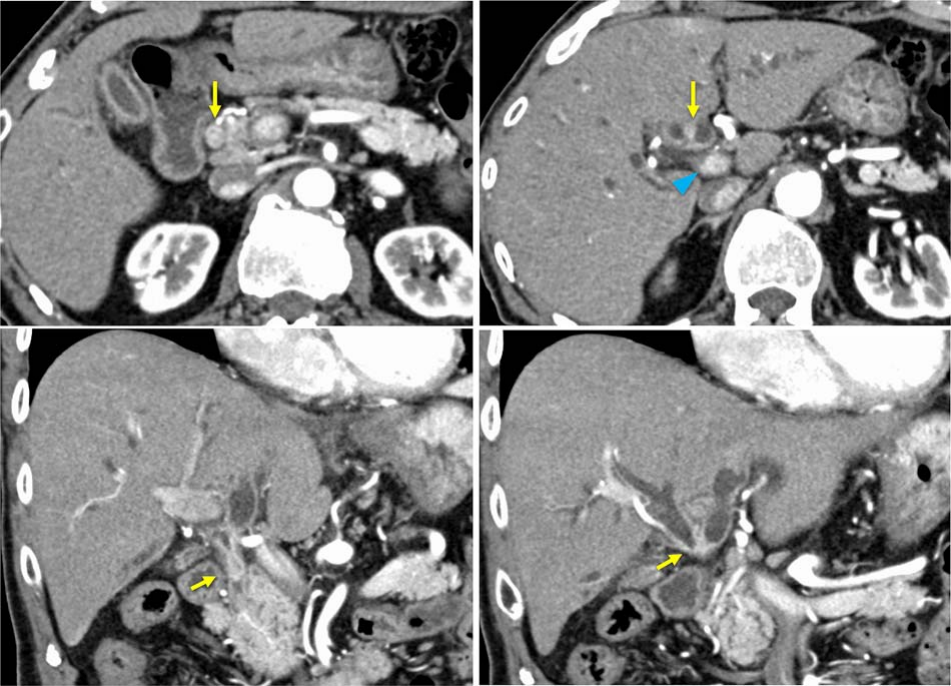
Preoperative contrast-enhanced computed tomography. Hilar bile duct thickening (arrows) and intrahepatic bile duct dilation are observed. The portal vein (arrowhead) is close to the thickened hilar bile duct.

A 76-year-old man was referred to our hospital due to fatigue, upper abdominal discomfort, and jaundice. He had no appreciable medical history. Multidetector row computed tomography revealed hilar bile duct thickening and intrahepatic bile duct dilation, suggestive of pCCA (Fig. 1).

Initial laboratory findings revealed elevated levels of hepatobiliary enzymes and C-reactive protein, but white blood cell and neutrophil counts were normal (Table 1). Obstructive jaundice without suppurative cholangitis was diagnosed. Given his thrombocytopenia (7.0 × 10^9^ /L) and elevated levels of platelet-associated immunoglobulin G (79 ng/10^7^ platelet), investigating the cause of the thrombocytopenia was a priority. Although the patient was positive for anti-*Helicobacter pylori* (*H. pylori*) antibodies, eradication was not performed immediately because of his low platelet count.

**Table 1:** Initial laboratory data upon admission

Laboratory data	Values
AST, IU/L	137
ALT, IU/L	114
T-bil, mg/dl	16.1
D-bil, mg/dl	12
ALP, IU/L	2270
BUN, mg/dl	13.6
Cre, mg/dl	0.9
TP, g/dl	7
Alb, g/dl	3.1
Na, mEq/L	135
K, mEq/L	3.6
Cl, mEq/L	101
CRP, mg/dl	6.18
WBC count, /μl	7230
Neut, %	86.1
Hb, g/dl	13.4
Plt, ×10^9^/L	7.0
PT-INR	1.35
Fib, mg/ml	566
FDP, μg/ml	4.6
PA IgG, ng/10^7^PLT	79
CEA, ng/ml	4.4
CA19–9, IU/ml	104.4
HBs-Ag	(−)
HBs-Ab	(+)
HBc-Ab	(+)
HCV-Ab	(−)
Anti-*Helicobacter pylori* Ab	(+)

Abbreviations: AST, aspartate aminotransferase; ALT, alanine aminotransferase; T-bil, Total bilirubin; D-bil, Direct bilirubin; ALP, Alkaline phosphatase; BUN, Blood urea nitrogen; Cre, Creatinine; TP, Total protein; ALB, Albumin; CRP, C-reactive protein; WBC, White blood cell; Neut, Neutrocyte; Hb, Hemoglobin; PLT, Platelets; PT-INR, Prothrombin time international normalized ratio; Fib, Fibrinogen; FDP, fibrinogen/fibrin degradation products; PAIgG, Platelet-associated IgG; CEA, Carcinoembryonic antigen; CA19–9, Carbohydrate antigen 19–9; HBs, Hepatitis B surface; HBc, Hepatitis B core; HCV, Hepatitis C virus; Ag, Antigen; Ab, Antibody, *H. pylori*; *H. pylori*.

**Figure 2:**
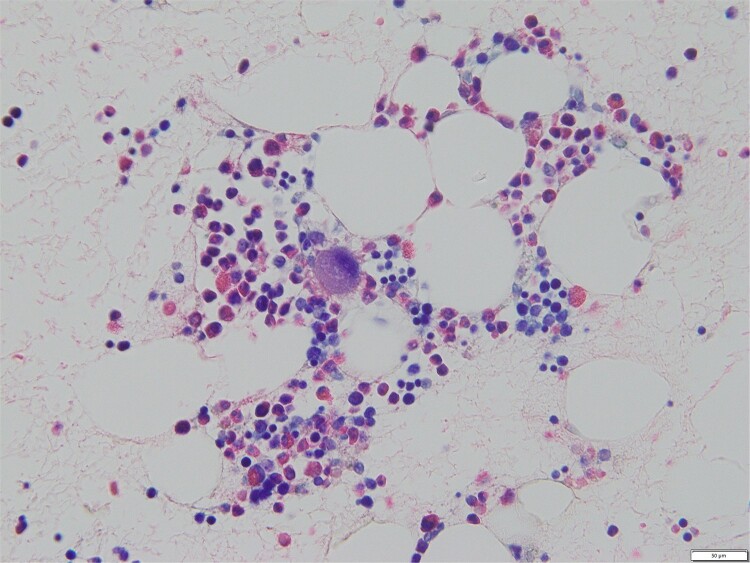
Bone marrow biopsy specimen (Naphthol AS-D chloroacetate esterase Giemsa staining, ×400). The myeloid/erythroblast ratio is normal, and the number of megakaryocytes is slightly increased without platelet adhesion.

**Figure 3:**
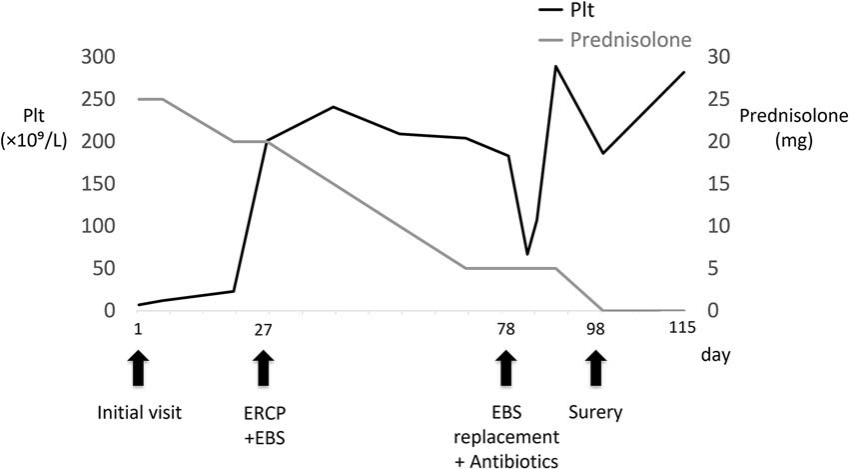
Time course, as well as trends of platelet counts and prednisolone dose. PLT, platelet count; ERCP, endoscopic retrograde cholangiopancreatography; EBS, endoscopic retrograde stent.

Bone marrow biopsy revealed a normal myeloid/erythroblast ratio and a slightly increased number of megakaryocytes without platelet adhesion (Fig. 2), consistent with ITP. The possibility of ITP secondary to pCCA was considered. Prednisolone therapy was initiated at 25 mg/day (0.5 mg/kg/day), with gradual dose reduction based on platelet response (Fig. 3). Lansoprazole 15 mg medication was also initiated for steroid ulcer prophylaxis.

**Figure 4:**
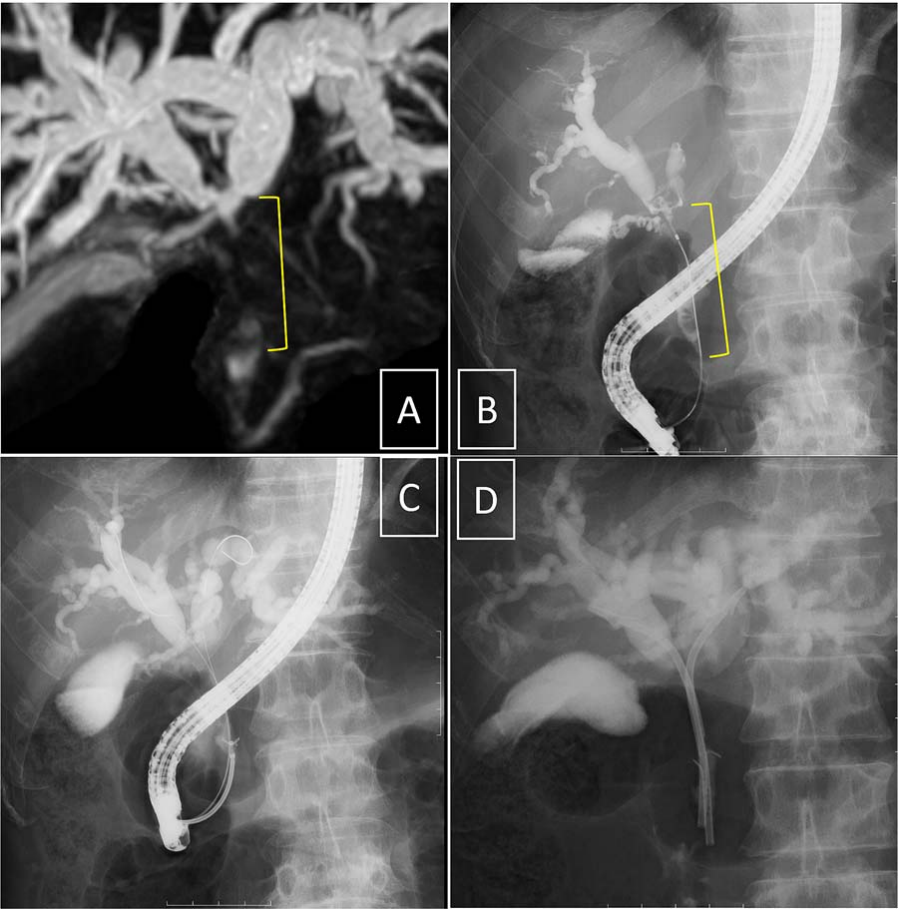
Cholangiography. Magnetic resonance imaging (A) and endoscopic retrograde cholangiopancreatography (B, D) reveal full circumferential stenosis in the mid-to-upper bile ducts without left and right bile disconnections. (C) The biopsy is conducted in the distal bile duct.

Endoscopic retrograde cholangiopancreatography (ERCP) was subsequently performed after the platelet count exceeded 50 × 10^9^ /L (perioperative Day 27), and an endoscopic biliary stent (EBS) was inserted. Cholangiography with magnetic resonance imaging and ERCP revealed full circumferential stenosis in the mid-to-upper bile ducts without left and right bile duct disconnections (Fig. 4). Intraductal ultrasonography further demonstrated superficial tumor involvement of the perihilar bile duct, without extension to the left or right hepatic ducts. Histological examination confirmed adenocarcinoma, consistent with Bismuth type II pCCA [8]. Tumor resection was planned when the Prednisolone dose was reduced to 5 mg/day, and the platelet count remained >100 × 10^9^ /L.

**Figure 5:**
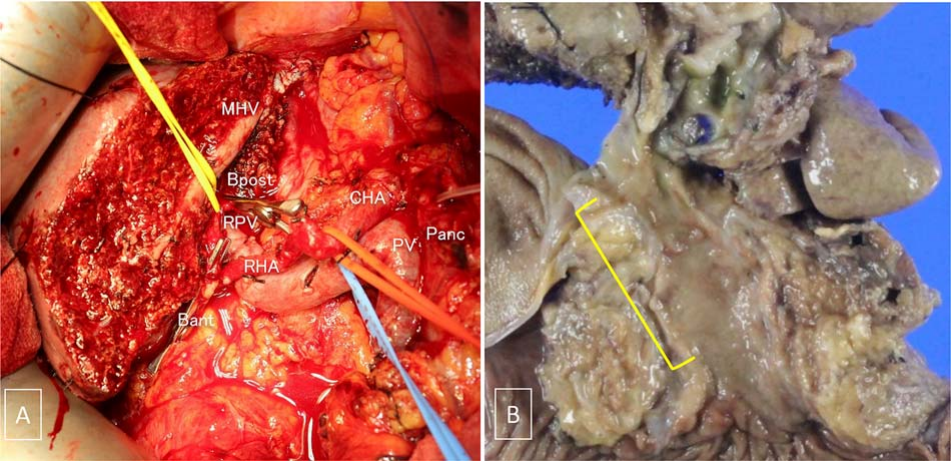
(A) Intraoperative photograph taken after tumor resection. (B) Gross examination of the resected specimen containing the tumor. MHV, the middle hepatic vein; Bpost, the right posterior branch of the bile duct; Bant, the anterior branch of the bile duct; PV, the portal vein; RPV, the right branch of the portal vein; CHA, the common hepatic artery; RHA, the right hepatic artery; Panc, pancreas.

On perioperative Day 78, the patient developed acute obstructive suppurative cholangitis, leading to a decrease in platelet count. Antibiotics (cefoperazone /sulbactam) and EBS replacement were effective in addressing this infection. Left hepatopancreatoduodenectomy with portal vein reconstruction was performed on perioperative Day 98. The bile duct and the portal vein were partially adherent, and the portion of portal vein was resected and reconstructed. The operative time was 399 min, and blood loss was 1300 ml, requiring two red blood cell units and 10 fresh frozen plasma units. Pathological examination confirmed well-differentiated tubular adenocarcinoma of the bile duct, flat-infiltrating type, T2aN1M0, Stage IIIC (UICC 8th edition [9]), with R0 resection (Fig. 5). Prednisolone therapy was discontinued postoperatively without ITP relapse. The patient was discharged on postoperative Day 22.

Adjuvant therapy with tegafur–gimeracil–oteracil potassium was initiated on postoperative Day 97 but was discontinued after one cycle due to fatigue. On postoperative Day 138, the patient was suspected to have lymph node recurrence and peritoneal dissemination; however, he was only followed-up due to an Eastern Cooperative Oncology Group performance status of 3. The patient unfortunately succumbed to cancerous peritonitis on postoperative Day 1088. Notably, ITP did not relapse during this period.

## Discussion

In the present case, the coexistence of ITP complicated the management of pCCA. The administration of prednisolone effectively improved platelet levels, allowing for the successful resection of pCCA via left hepatopancreatoduodenectomy. Remarkably, following surgery, the patient went into remission for ITP without the need for continued Prednisolone therapy, and importantly, no relapse was observed thereafter.

pCCA is a rare biliary tract malignancy (0.74–1.05 per 100 000 people), with surgery as the only curative treatment [10, 11]. Unfortunately, most patients with pCCA are diagnosed at advanced stages, requiring extensive surgical procedures, such as hepatic trisectionectomy, hepatectomy with pancreaticoduodenectomy, and combined vascular resection with reconstruction [12]. Generally, these procedures require a minimum platelet count of 80 × 10^9^/L to avoid uncontrolled intraoperative bleeding [3].

Conversely, ITP, characterized by platelet counts <100 × 10^9^/L [1], poses significant challenges in surgical management due to increased risks of bleeding. Particularly, fatal bleeding has been reported in adults with ITP when platelet counts fall <30 × 10^9^/L [1]. As previously mentioned, ITP can be categorized as primary or secondary [3]. Various underlying diseases can trigger secondary ITP, including malignancies, among which lung and breast cancers are the most common. However, no studies have reported that pCCA may also trigger secondary ITP [13]. Despite the lack of definitive diagnostic criteria for such cases, the absence of ITP relapse following pCCA resection strongly suggests a secondary etiology. Incidentally, the present case showed positivity for anti-*H. pylori* antibodies and may have been infected with *H. pylori*. Eradication was not recommended when platelet levels were < 10 × 10^9^ /L [14], but should have been performed once the platelet count improved. Eventually, it was not considered to be *H. pylori*-associated ITP because of its remission without eradication.

Corticosteroids remain the mainstay of ITP treatment [15]. However, ~80% of patients develop corticosteroid dependence [4], and its long-term use carries the risk of adverse effects. Interestingly, several case reports have documented successful remission following tumor treatment in patients with secondary ITP [5–7], underscoring the potential benefits of addressing underlying malignancies.

In conclusion, prompt diagnosis and Prednisolone treatment enabled successful pCCA resection, emphasizing the critical role of platelet management in surgical outcomes. Achieving ITP remission postoperatively prevented both the risk of life-threatening bleeding and the need for prolonged Prednisolone therapy. This case report demonstrates the significance of integrated management strategies in complex cases, allowing for the optimization of patient outcomes.

## Data Availability

Data sharing is not applicable to this article as no datasets were generated or analysed during the current study.
